# The First Symbiont-Free Genome Sequence of Marine Red Alga, Susabi-nori (*Pyropia yezoensis*)

**DOI:** 10.1371/journal.pone.0057122

**Published:** 2013-03-11

**Authors:** Yoji Nakamura, Naobumi Sasaki, Masahiro Kobayashi, Nobuhiko Ojima, Motoshige Yasuike, Yuya Shigenobu, Masataka Satomi, Yoshiya Fukuma, Koji Shiwaku, Atsumi Tsujimoto, Takanori Kobayashi, Ichiro Nakayama, Fuminari Ito, Kazuhiro Nakajima, Motohiko Sano, Tokio Wada, Satoru Kuhara, Kiyoshi Inouye, Takashi Gojobori, Kazuho Ikeo

**Affiliations:** 1 National Research Institute of Fisheries Science, Fisheries Research Agency, Yokohama, Kanagawa, Japan; 2 Center for Information Biology, National Institute of Genetics, Mishima, Shizuoka, Japan; 3 Seikai National Fisheries Research Institute, Fisheries Research Agency, Nagasaki-shi, Nagasaki, Japan; 4 Hitachi Solutions, Ltd., Shinagawa-ku, Tokyo, Japan; 5 Japan Software Management Co. Ltd., Yokohama, Kanagawa, Japan; 6 Fisheries Research Agency, Yokohama, Kanagawa, Japan; 7 Ministry of Agriculture, Forestry and Fisheries, Chiyoda-ku, Tokyo, Japan; 8 National Research Institute of Aquaculture, Fisheries Research Agency, Minami-ise, Mie, Japan; 9 Japan Sea National Fisheries Research Institute, Fisheries Research Agency, Chuou-ku, Niigata, Japan; 10 Division of Molecular Biosciences, Department of Bioscience and Biotechnology, Faculty of Agriculture, Kyushu University, Higashi-ku, Fukuoka, Japan; French National Centre for Scientific Research, Université Paris-Sud, France

## Abstract

Nori, a marine red alga, is one of the most profitable mariculture crops in the world. However, the biological properties of this macroalga are poorly understood at the molecular level. In this study, we determined the draft genome sequence of susabi-nori (*Pyropia yezoensis*) using next-generation sequencing platforms. For sequencing, thalli of *P. yezoensis* were washed to remove bacteria attached on the cell surface and enzymatically prepared as purified protoplasts. The assembled contig size of the *P. yezoensis* nuclear genome was approximately 43 megabases (Mb), which is an order of magnitude smaller than the previously estimated genome size. A total of 10,327 gene models were predicted and about 60% of the genes validated lack introns and the other genes have shorter introns compared to large-genome algae, which is consistent with the compact size of the *P. yezoensis* genome. A sequence homology search showed that 3,611 genes (35%) are functionally unknown and only 2,069 gene groups are in common with those of the unicellular red alga, *Cyanidioschyzon merolae*. As color trait determinants of red algae, light-harvesting genes involved in the phycobilisome were predicted from the *P. yezoensis* nuclear genome. In particular, we found a second homolog of phycobilisome-degradation gene, which is usually chloroplast-encoded, possibly providing a novel target for color fading of susabi-nori in aquaculture. These findings shed light on unexplained features of macroalgal genes and genomes, and suggest that the genome of *P. yezoensis* is a promising model genome of marine red algae.

## Introduction

Marine red algae of the order Bangiales (Rhodophyta) such as *Pyropia* and *Porphyra* (laver) have been important seafoods in East and Southeast Asia for thousands of years [1]. Lavers are also harvested in New Zealand, Chile, Wales, and Pacific North America [2]–[6]. In Japan, the aquaculture of Bangiales seaweeds (so-called ‘nori’) started three hundred years ago, and many different species have been cultivated. Currently, susabi-nori (*Pyropia yezoensis*) is the highly valued seaweed crop in East Asia, as well as in Japan.

In terms of the morphogenesis of nori, their life history remained enigmatic, even after the aquaculture became popular. Bangiales seaweeds were usually harvested as macrophytes of thalli in winter, but little was known about their morphological characters in other seasons. The question was resolved by Drew’s works [7], [8], which showed that gametophytes alternate with a microscopic, filamentous, shell-boring sporophytic phase. This milestone accelerated research into the cell biology of nori, providing the basis for understanding how the organisms live in the seawater environment. Over the last decade, host-symbiont interaction has been a hot topic of research into the life history of seaweed. Many seaweeds contain the essential coenzyme vitamin B_12_ (cobalamin), and this metabolite is often supplied from environmental bacteria [9]. *P. yezoensis* also contains high levels of vitamin B_12_, and bacteria are considered the source. Although there are many kinds of marine bacteria on the cell surface of *P. yezoensis* [10], some of which are tightly attached to the cell wall, their biological significance is still uncertain. A recent study demonstrated that some of these bacteria play key roles in the normal development of *P. yezoensis* (unpublished data), suggesting that there is a symbiotic relationship between them.

In the field of aquaculture, cultivar improvement of *P. yezoensis* has been an important issue. Color fading, for example, is a disease of *Pyropia* caused by nutrient deficiency in water, and there have been efforts to clarify the mechanism and develop resistant cultivars. Climate change, such as global warming, has also raised concerns about nori aquaculture. Many of the current cultivars originate from the northern cold area, and their tolerance to higher temperatures has been examined. Recently, breeding and molecular cloning technologies have enabled the development of DNA markers for *Pyropia*. However, species of Bangiales have simple morphological features, making it difficult to distinguish them from each other. Therefore, the genome sequence of *Pyropia* will be a promising resource for the comprehensive development of high-resolution markers.

Among red algae, the 100%-complete genome sequence of a unicellular species, *Cyanidioschyzon merolae*, has been determined [11], [12]. This organism inhabits extreme environments, such as hot springs, and may be a target of special adaptation, though the genome is expected to provide basic information on the lifestyle of photosynthetic eukaryotes. As another special case, the nucleomorphs in cryptomonads cells, which are considered to be of red-algal symbiont origin, have been also sequenced [13]–[15]. The nucleomorph genomes are generally quite small (several hundred kilobase pairs) and have lost many genes, indicating that genome reduction or gene transfer to the host nucleus have occurred after symbiosis. The nuclear DNA contents of Bangiales species have been examined by fluorescence microscopy [16]–[18], and range from 260 to 500 Mb, but this has not been confirmed at the sequence level. *P. yezoensis* has been considered a good target for red algal genomics [19], and expressed sequence tag (EST) analyses have been conducted to explore the gene candidates related to the life cycle [20]–[22]. However, the whole genome sequencing of this marine alga has been difficult because of DNA contamination from symbiotic bacteria. Thus, for red algae in general, genomic information has been poor until now, and many of the molecular mechanisms related to their life cycle or other traits remained unsolved. In this study, we have prepared axenic protoplast culture of *P. yezoensis*, sequenced the nuclear genome, and report its genomic features.

## Materials and Methods

### Protoplast isolation from *P. yezoensis* Culture

Monospores of *P. yezoensis* strain U-51 were cultured in sterile modified half-strength SWM-III medium. The culture was incubated at 17°C under illumination (50 µmol·m^−2^·s^−1^, 10∶14 h light:dark cycle). The culture medium was replaced every week. For the isolation of protoplasts, samples of the formed thalli were harvested directly from the culture flasks. The isolation procedure was according to a previously modified method [23]. In brief, the thalli weighing about 50–100 mg were immersed in 0.5% citric acid (pH 2.0–2.3) for 90 s and rinsed with sterile 90% natural seawater (NSW). The cleaned thalli were cut with a microtome blade and shaken with a 2% papain solution for 30 min. After washing with 90% NSW containing 0.7 M mannitol, enzyme solutions of agarase, mannanase, and xylanase (1 unit/8 ml, Yakult pharmaceutical industry Co., LTD, Tokyo, Japan) were added to the thalli and shaken for 60–90 min to degrade the cell walls. The solution was filtered through 20-µm mesh filter to remove the undigested tissue debris. The filtrate fraction was collected as the cell wall sample. The filtered solution was washed with 90% NSW containing 0.7 M mannitol, and the protoplast solution was obtained.

### Genome Sequencing and Assembly

From the protoplast DNA sample of *P. yezoensis*, whole-genome shotgun libraries were prepared for two platforms, a Roche 454 GS-FLX/FLX+ (Roche Diagnostics, Branford, CT) and an Illumina Genome Analyzer IIx (Illumina, Inc. San Diego, CA), respectively. The 454-pyrosequencing library for single-end reads was constructed from the sheared DNA by GS Titanium Rapid Library Preparation Kit (Roche Diagnostics). For the Illumina Genome Analyzer IIx, a 75-bp paired-end shotgun library (insert sizes of 500 bp) was prepared according to the manufacturer's protocols. The read data obtained have been deposited in DDBJ/EMBL/GenBank under accession number SRA061934. Both reads were assembled using CLC Assembly Cell™ version 4.06 beta (CLC bio, Aarhus N, Denmark). For the preliminary *de novo* assembly, the contigs obtained still contained the sequences of organelles (mitochondrion and chloroplast) and an unknown bacterium of the genus *Agarivorans*. To remove such non-nuclear sequences, reference sequences were prepared. For organelles, the sequences of the chloroplast (accession: NC_007932) and mitochondrion (NC_017837) genomes of *P. yezoensis* were downloaded from the GenBank. In addition, *Agarivorans albus* strain MKT 106 [24] was purchased from the National Institute of Technology and Evaluation, Japan (NBRC) and the genomic sequences were read with 454 GS FLX+ (Text S1). The sequences of the organelles and bacterium were then used to clean the assembly of *P. yezoensis* nuclear DNA sequences.

### cDNA Sequencing

Total RNA was isolated from thalli of *P. yezoensis* U-51 and first-strand cDNA was synthesized to selectively enrich full-length one with a cap structure and polyA tail (Text S2). The cDNA library prepared was sequenced by Illumina Genome Analyzer IIx, and finally 12,570,945 read pairs were obtained.

### Detection of Transposable Elements and Repetitive Sequences

Tandem repeats in the *P. yezoensis* nuclear genome were detected by Tandem Repeats Finder version 4.04 program [25] with default parameters, followed by analysis using Tandem Repeats Analysis program version 2.1 [26]. The resulting 6,300 consensus sequences were employed to build repeat library generated by RepeatScout version 1.0.5 [27]. Knowledge-based annotation was carried out by REPET pipeline (http://urgi.versailles.inra.fr/Tools/REPET) [28]. Transposons and SINEs (short interspersed nuclear elements) were identified using CENSOR software version 4.2 [29].

### Gene Prediction

We downloaded the known protein sequences of red algae (Rhodophyta) from GenBank at NCBI. We mapped those to the genome contigs assembled and detected 547 coding regions using Exonerate [30]. To detect other coding regions in the contigs, we prepared a training model using the gene-finding program, AUGUSTUS [31]. The initial training model was constructed using the 547 nucleotide sequences, and 7,733 genes were predicted from the contigs. We then queried the predicted protein sequences against the NCBI *nr* database using BLASTP (E-value <1e-10) [32], and appended 382 well-matched queries to the training model. Thus, we reconstructed the training model with 929 nucleotide sequences in total. During gene prediction, 12,570,945 cDNA read pairs in this study and 28,465 EST sequences of *Pyropia* from GenBank were mapped to the contigs by BLAT [33] and utilized as hints of exon regions in AUGUSTUS. Algal and plant protein sequences from the public databases (the species and sources are listed in Table S1) were mapped by TBLASTN (E-value <1e-5) and also hinted for AUGUSTUS. Predicted gene sequences are available at http://nrifs.fra.affrc.go.jp/ResearchCenter/5_AG/genomes/nori/.

### Gene Annotation and Comparison

Each gene function was inferred by BLASTP (E-value <1e-5) to the GenBank database and mapping to the Gene Ontology (GO) was conducted by Blast2GO [34]. Protein sequences from red/green algal and plant genomes, *C. merolae*, *Chlamydomonas reinhardtii* and *Arabidopsis thaliana* (Table S1), were used for gene set comparison. Orthologous gene pairs were defined as reciprocal best hit pairs by BLASTP (E-value <1e-5). The proportions of GO categories annotated by Blast2GO among species were compared using WEGO [35].

### Analyses of Methionine Synthase Genes and Light-harvesting Genes

Multiple sequence alignment and phylogenetic analysis were conducted by MAFFT [36] and MEGA5 [37], respectively. Phylogenetic trees were constructed by the Neighbor-Joining method [38]. The subcellular localization of light-harvesting proteins to chloroplast were predicted by WoLF PSORT [39] and ChloroP 1.1 [40]. For an NblA homolog of *P. yezoensis*, the cDNA was synthesized from total RNA described above and PCR experiment was conducted to confirm the gene structure and expression (Text S3).

## Results

### Assembly and Genome Statistics

The Roche 454 GS FLX and the Illumina GA IIx platforms generated 1,810,613 reads with 9,474,994,049 base pairs (bp) and 508,033,476 reads with 254,016,738 base pairs, respectively. Based on read coverage analysis, the whole sequence of nearly 510 million reads was estimated to represent an approximately 166-fold coverage of the *P. yezoensis* nuclear genome (Figure S1). During the assembly, we removed non-nuclear contigs such as organellar and bacterial sequences, finally obtaining 46,634 contigs (Table 1). The contigs totaled 43 Mb with a high GC content (63.6%), while the organellar genomes have a low GC% (mitochondrion; 32.7% and chloroplast; 33.1%). The average contig length and N50 were 932 bp and 1,669 bp, respectively, indicating that the genome sequences are still fragmented. About 98% (12,309,453 read pairs) of cDNA read pairs were mapped on the contigs. In addition to the nuclear genome, we found two closed-circular plasmids similar to those of *Pyropia tenera* (Text S4).

**Table 1:** Assembly statistics of the *P. yezoensis* genome.

Total contig size (bp)	43,483,963
Number of contigs	46,634
Average contig length (bp)	932
Contig N50 (bp)	1,669
Contig coverage	166
Percentage of mapped cDNA read pairs (%)	97.9
G+C content (%)	63.6
Percentage of repetitive sequences (%)	1.4

### Repetitive Sequences and Telomere Structure

**Figure 1:**
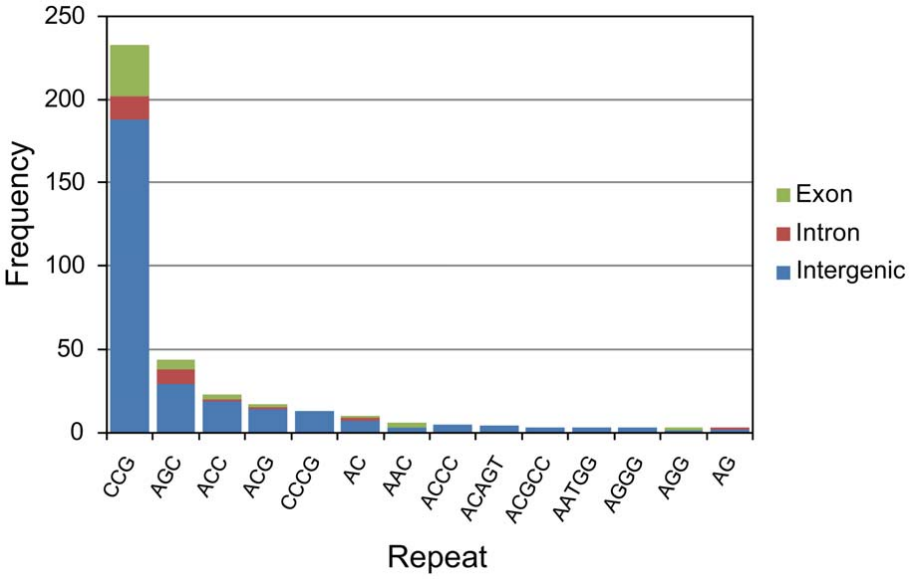
Microsatellite distribution. Repeats with frequency > = 3 are shown. Locations of microsatellite repeats are classified by color: exon (green), intron (red) and intergenic (blue) regions.

In the nuclear genome, there are only 392 microsatellite (2 to 5-bp) repeats, most of which are of 3-bp (326 repeats) (Figure 1). In particular, the majority of triplet repeats (233/326) are CCG (i.e., CCG/CGC/CGG/GCC/GCG/GGC) and are generally located in intergenic regions. The paucity of short tandem repeats is comparable to the *C. merolae* genome, which has 569 repeats, mostly AGC triplets (Figure S2). Non-redundant repetitive sequences make up 1.4% of all the contigs (Table1). We did not find telomere sequences in the assembled contigs, but did in the paired-end reads produced by Illumina. The unit of the telomere was estimated to be 5′-TTAGGG-3′, similar to those of land plants and green algae (5′-TTTAGGG-3′) [41].

### Gene Statistics

**Figure 2:**
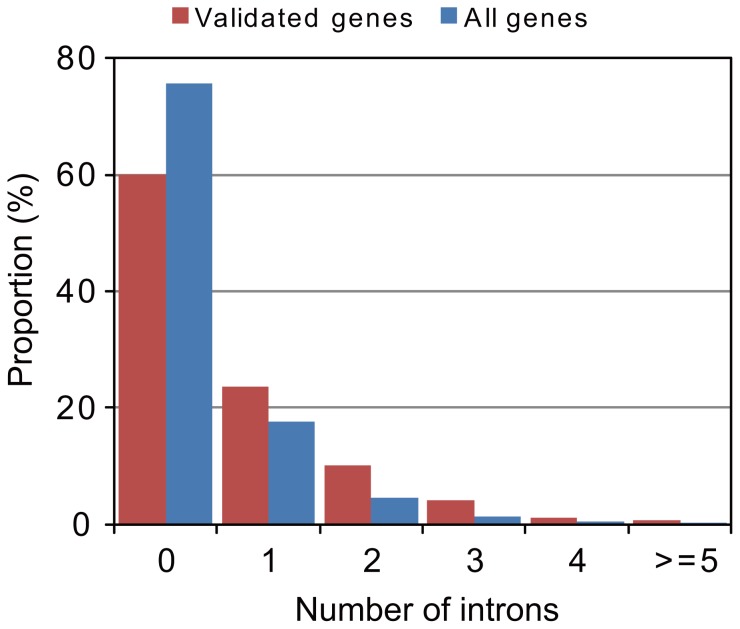
Distribution of intron number. “All genes” and “Validated genes” correspond to those shown in Table 2, respectively.

**Figure 3:**
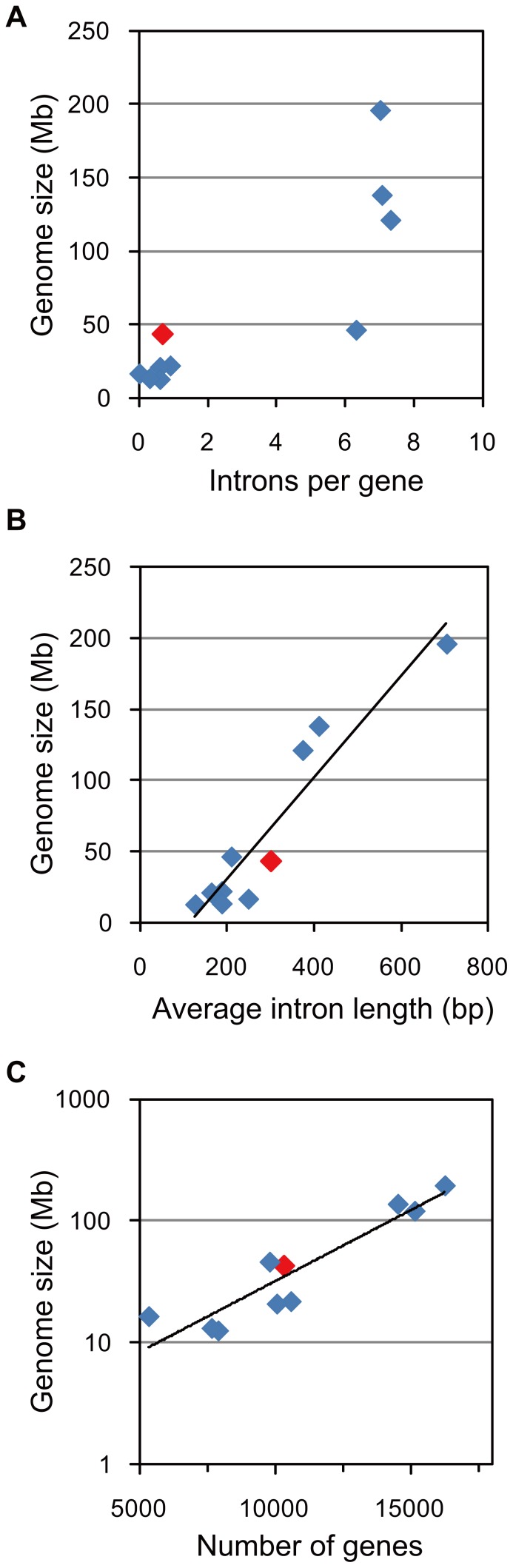
Correlation between gene statistics and algal genome sizes. The *y* value of *P. yezoensis* (plotted in red) indicates the contig size (43 Mb). The species and data that are not shown in Table 2 are summarized in Table S2. (A) Intron density and genome size; (B) Average intron length and genome size; (C) Gene content and (logarithmic-transformed) genome size.

We predicted 10,327 protein-coding gene models from the nuclear genome (Table 2), which is about twice as many as *C. merolae* [11]. In comparison with other algal genes, the average CDS length was short (849 bp), probably because many of the genes are partially predicted from fragmented contigs. Therefore, to obtain robust statistics of *P. yezoensis* genes, we selected 1,314 genes as validated, each protein sequence of which covered more than 95% of the homologous sequence in an alignment. This produced an increased average CDS length (1,247 bp), which is comparable to those of other algae. In particular, we found that the genes of *P. yezoensis* had few introns: about 60% of the validated genes lacked introns (Figure 2). As for all predicted genes, the genes lacking introns made up nearly three quarters of those. The average number of introns per gene (0.7/gene) is similar to those of small-genome algae (Figure 3A), and is 10-fold lower than that of *Chlorella variabilis* NC64A, a green alga with a similar genome size (46 Mb) [42]. The average intron size was also small (300 bp) compared with large-genome algae, such as a green alga *C. reinhardtii* [43] and a brown alga *Ectocarpus siliculosus* [44]. We observed a correlation between average intron length and algal genome/contig size (Figure 3B), as well as between gene number and genome/contig size (Figure 3C).

**Table 2:** Characteristics of *P. yezoensis* genes.

Species	*P. yezoensis*		*C. merolae*	*C. variabilis* NC64A	*C. reinhardtii*	*E. siliculosus*
Genome or contig size (Mb)	43		16.5	46.2	121	195.8
	All genes predicted	Validated genes				
Gene content	10,327	1,314	5,331	9,791	15,143	16,256
Gene density (kb/gene)	4.2	–	3.1	4.7	8.0	12.0
Average CDS length (bp)	849	1,247	1,552	1,371	1,335	1,563
Average exon legth (bp)	634	755	1,540	170	190	242
Average intron length (bp)	304	300	248	209	373	704
Introns per gene	0.3	0.7	0.005	6.3	7.3	7.0

### Gene Annotation and Comparison

**Figure 4:**
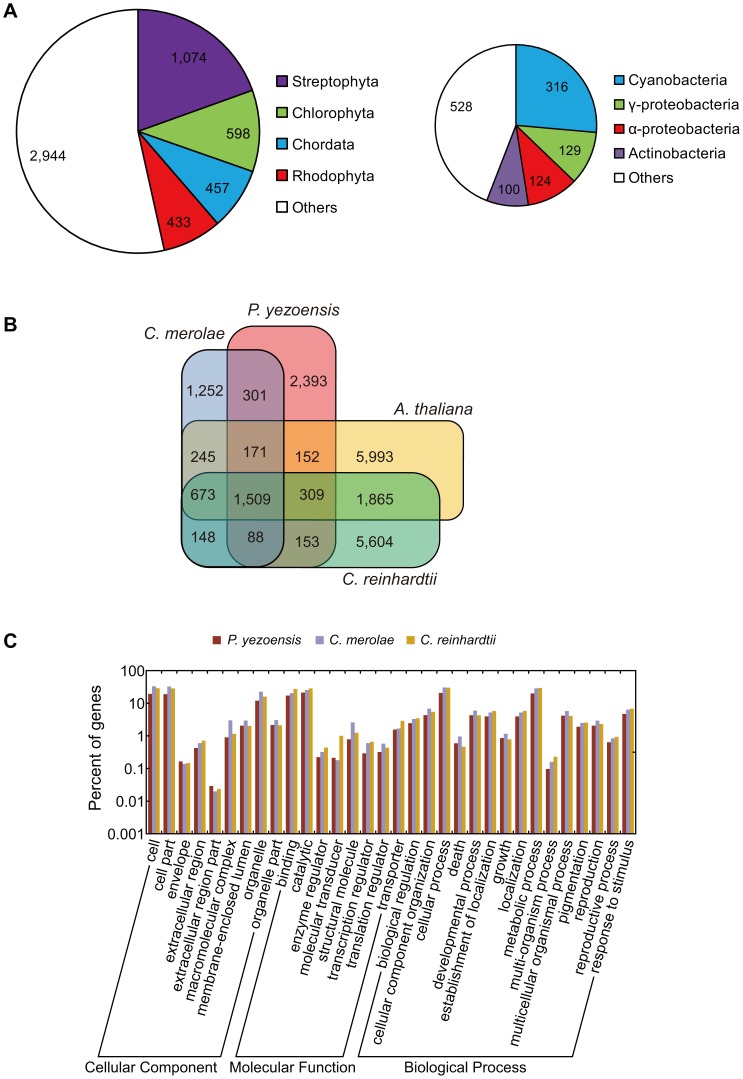
BLAST top hit distribution and gene set comparison. (A) Taxonomic distributions of BLASTP top hits of *P. yezoensis* genes. (left) Eukaryotes; (right) Prokaryotes; (B) A Venn diagram of gene sets among four species (*P. yezoensis*, *C. merolae*, *C. reinhardtii*, and *A. thaliana*). Numbers of gene groups are shown on the diagram. Each gene group is defined as a singleton or a cluster of paralogs; (C) GO category comparison among *P. yezoensis*, *C. merolae* and *C. reinhardtii*.

Of the 10,327 genes predicted in the *P. yezoensis* nuclear genome, 6,716 genes matched known genes in GenBank and 3,611 were novel. About 80% of the BLAST top hits were to other eukaryotes: the Streptophyta (mainly land plants) genes account for the majority (Figure 4A, left), followed by Chlorophyta (green algae), Chordata (e.g. vertebrates), and Rhodophyta (red algae). This distribution probably reflects the proportion of entries in GenBank, because many land plant and vertebrate genomes have been sequenced. The remaining 20% of the top hits were to bacteria (Figure 4A, right), many of which were from cyanobacteria, which may have been transferred from the chloroplast genome. Similarly, α-proteobacterial hits might be accounted for by the transfer from an ancestral genome of the mitochondrion. The other hits might be examples of gene transfer from environmental species, or represent artifacts from deeply diverged sequences. In this study, a case of horizontal gene transfer from γ-proteobacteria was suggested by Sanger resequencing (Figure S3).

Rhodophyta are phylogenetically close to Viridiplantae (Chlorophyta and Streptophyta) [45]; therefore, we selected the representative model species, *C. merolae*, *C. reinhardtii* and *A. thaliana*, and compared the gene sets among those three and *P. yezoensis*. As a result, 1,509 gene groups were common to *C. merolae*, *C. reinhardtii* and *A. thaliana* (Figure 4B). These groups are composed of 1,813 genes of *P. yezoensis*, 1,610 genes of *C. merolae*, 1,762 genes of *C. reinhardtii*, and 3,237 genes of *A. thaliana*, respectively (Figure S4), indicating that many of the algal core genes are singletons. We detected 3,946 ( = 1,252+301+2,393) Rhodophyta-specific groups, and 2,393 of these were found in only *P. yezoensis*, but not in *C. merolae*. There were 2,069 ( = 301+171+1,509+88) orthologous gene groups between the two red algae: 2,059 between *P. yezoensis* and *C. reinhardtii*, and 2,141 between *P. yezoensis* and *A. thaliana*. There are slightly more gene groups missing only from *P. yezoensis* (673 groups) than the other lineages, but the proportion of GO categories does not show marked differences from other algae (Figure 4C).

**Figure 5:**
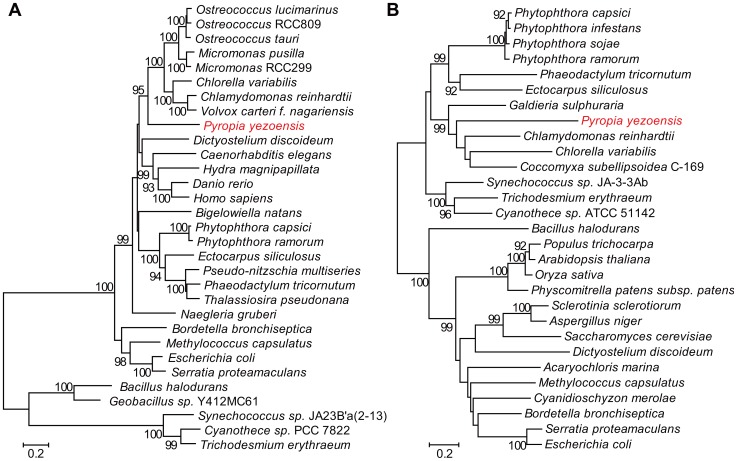
Phylogenetic relationships of METH and METE. Nodes with bootstrap probabilities > = 90% (1000 replicates) are shown. (A) Phylogenetic tree for METH and (B) Phylogenetic tree for METE. The accession numbers of sequences compared are summarized in Table S3.

We identified two types of genes encoding methionine synthase: vitamin B_12_-dependent (METH) and independent (METE), in the *P. yezoensis* genome. To the best of our knowledge, this is the first identification of a red algal METH. Phylogenetic analysis showed that each of the proteins formed a sister clade to those of green algae, such as *Chlamydomonas* (Figure 5). In the tree of METE, the *P. yezoensis* protein was positioned near to that of another red alga *Galdieria sulphuraria*, but distant from that of *C. merolae*. Thus, it is likely that both of the *P. yezoensis* methionine synthase genes have originated in the lineage of red algae and green algae. We did not found any vitamin B_12_ biosynthesis genes, except for cobalamin adenosyltransferase and cobalamin 5′-phosphate synthase, among the current contigs, implying that *P. yezoensis* utilizes the vitamin via symbiotic bacteria, like many algae [9].

### Photosynthesis Genes in the Nuclear Genome

**Figure 6:**
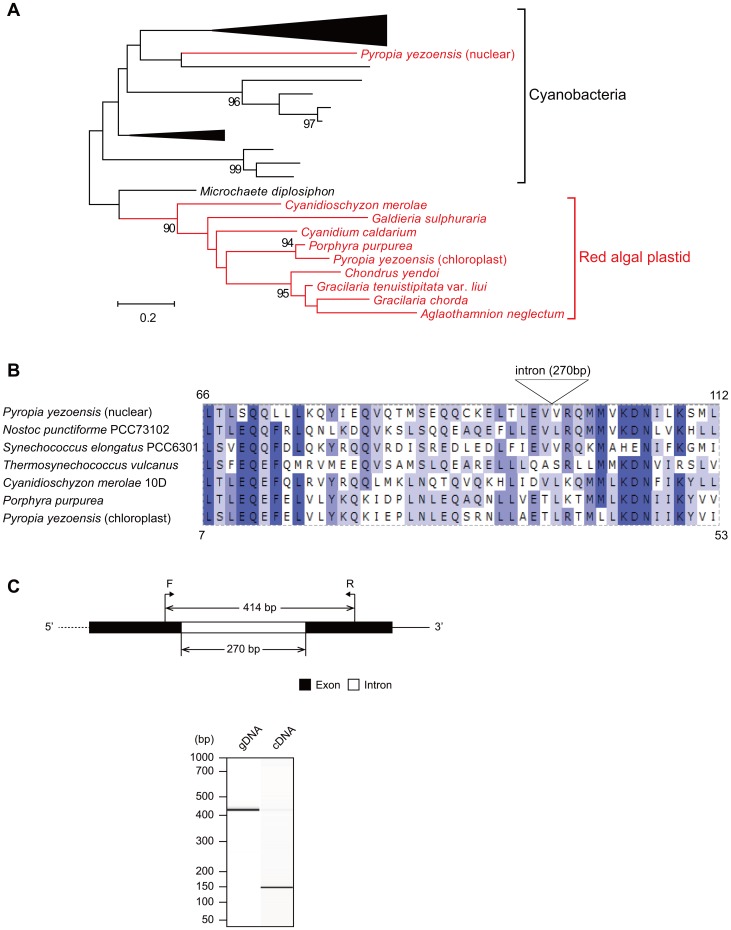
Structure of the *NblA* locus and sequence analysis. (A) A phylogenetic tree of NblA proteins. Nodes with bootstrap probabilities > = 90% (1000 replicates) are shown. Red algae are indicated in red and cyanobacteria are indicated in black. The accession numbers of sequences compared are summarized in Table S4; (B) A partial alignment of NblA proteins. More highly conserved residues are shown in deeper blue. The numbers in the corners indicate alignment start/end positions of amino acid residues in the *P. yezoensis* nuclear/plastid NblA homologs, respectively; (C) Predicted genomic structure and PCR amplification of *NblA* locus. The position of forward (F) and reverse (R) primers used is indicated by arrows on the predicted genomic structure. PCR amplification of the gene was performed using genomic DNA (gDNA) from protoplasts and complementary DNA (cDNA) from thalli of *P. yezoensis* as templates. The dotted line in the genomic structure represents undetermined nucleotide sequence.

From the lineage-specific genes detected above, we surveyed those involved in photosynthesis, which may control color traits of *P. yezoensis*. From the nuclear genome, we indentified light-harvesting genes that were unreported in *P. yezoensis* (Table 3). All of the genes have high GC content (average 66.5%), which is close to the average of nuclear genes but not plastid genes. Most of these proteins were predicted to be localized in the chloroplast, suggesting that they actually function in photosynthesis. In this study, phycobilisome-related genes, such as an R-phycoerythrin γ-subunit gene, were mainly predicted rather than chlorophyll-related genes. In particular, we found two types of genes that have never been reported in red algae: phycobilisome linker proteins and phycobilisome-degradation protein (NblA) that match to cyanobacterial homologs. Previously, an *NblA*-like gene, *Ycf18*, was identified near to phycoerythrin α- and β-subunit genes in the chloroplast genome of *P. yezoensis* [46], but another *NblA* homolog in the nuclear genome was not as similar to *Ycf18* as it was to cyanobacterial homologs. In the phylogenetic analysis, we did not obtain a robust tree because the NblA sequences are too short (∼60 aa) (Figure 6A). However, the topology apparently shows that the nuclear *NblA* homolog of *P. yezoensis* has evolved in a different manner from the red-algal-plastid *NblA* homologs. The nuclear *NblA* homolog is predicted to comprise two exons, both of which have conserved regions with the known homologs (Figure 6B). This gene structure, mRNA expression and splicing were confirmed experimentally (Figure 6C). Therefore, we now propose that the nuclear NblA is active in *P. yezoensis* cells, although the subcellular localization is not predicted to be the chloroplast.

**Table 3:** Light-harvesting genes in the *P. yezoensis* nuclear genome.

Gene ID*	Top hit accession	Top hit species	Description	Identity (%)	E-value	G+C%	WoLF PSORT	ChloroP
**Phycobilisome-related genes**								
g2786	AAP59423	*Corallina officinalis*	R-phycoerythrin gamma subunit	47.3	4E-83	64.4	✓	✓
g5483	AAB37302	*Aglaothamnion neglectum*	gamma 31 kDa subunit of phycoerythrin precursor	37.5	4E-35	70.4	✓	✓
g2698	ZP_10229441	*Microcystis* sp. T1–4**	Phycobilisome 27.9 kDa linker polypeptide, phycoerythrin-associated, rod	49.7	5E-42	65.5	✓	✓
g2303	AAP80724	*Griffithsia japonica*	phycobilisome 31.8kD linker polypeptide	61.1	2E-107	64.0	✓	✓
g8704	YP_001521651	*Acaryochloris marina* MBIC11017**	phycobilisome 32.1 kDa linker polypeptide	41.6	4E-28	66.6	✓	✓
g9334	AAP80835	*Griffithsia japonica*	phycobilisome 7.8 kDa linker polypeptide	64.6	1E-35	65.0	✓	✓
g5407	ZP_08427732	*Lyngbya majuscula* 3L**	phycobilisome linker polypeptide	46.0	4E-30	68.5	✓	✓
g4291	NP_924210	*Gloeobacter violaceus* PCC 7421**	phycoerythrin-associated linker protein	52.1	4E-40	65.9	✓	✓
g3612	YP_001867135	*Nostoc punctiforme* PCC 73102**	phycobilisome degradation protein NblA	47.2	1E-07	65.0		
**Chlorophyll-related genes**								
g3715	ZP_06307232	*Cylindrospermopsis raciborskii* CS-505**	chlorophyll synthetase	65.65	6E-154	66.8	✓	✓
g6733	CBN79181	*Ectocarpus siliculosus*	Protochlorophyllide reductase, putative chloroplast precursor	54.86	7E-110	69.5	✓	

* Numbered based on AUGUSTUS prediction.

** Cyanobacteria.

## Discussion

During the sequencing of the *P. yezoensis* genome, a potentially serious problem was DNA sampling. As reported previously, many bacterial species are deeply attached to *P. yezoensis* cells and form a sort of biofilm, which could result in contamination during genome sequencing. In this study, we cultured axenic protoplasts of *P. yezoensis* to remove such symbiotic or attached bacteria and prepared the purified nuclear DNA. Since we found that some of the contigs were still of organellar (chloroplast and mitochondrion) and bacterial sequences in the pilot genome assembly, we removed these contigs by reference sequecnes. After removing non-nuclear contigs, we finally obtained 46,634 contigs for the *P. yezoensis* genome, spanning approximately 43 Mb. A plausible explanation of the contig’s fragmentation is that the nuclear genome has a high GC content, whereby next-generation sequencers read through chromosomal regions [47]. The contig coverage of 166-fold redundancy is nonetheless enough to sketch the genome features of *P. yezoensis*. One of the implications is that the total contig size was unexpectedly compact. It is commonly argued that nuclear DNA contents of *Pyropia* species range from 260 Mb of *P. yezoensis* [18] to 500 Mb [17], and our estimate is an order of magnitude smaller than those. This discrepancy does not seem to be explained only by genomic GC content bias. In a previous study, GC-rich sequences uncovered by Illumina did not exceed 10% of the whole genome [48]. Therefore, we propose that the genome of *P. yezoensis* is actually smaller than previously imagined. The evidence for a compact genome may also be provided by comparison of introns among algal genomes. While exon regions may be influenced by gene functional constraints, introns are considered relatively neutral and their lengths probably reflect genome expansion/reduction tendencies [49]. Actually, the average intron length of *P. yezoensis* was smaller than those of *C. reinhardtii* and *E. siliculosus*. In particular, we observed a linear correlation between the average intron length and genome size, which may be applicable for estimating the DNA contents of unfinished algal genomes. Although the intron density is not linearly correlated with genome size, that of *P. yezoensis* (0.7 per gene) is a feature of small-genome algae. In addition, a lower frequency of repetitive sequences including microsatellite repeats might be involved in the compact genome structure of *P. yezoensis*.

In this study, we predicted 10,327 gene models from the nuclear genome of *P. yezoensis*. One caveat to this estimate is that many of the genes are partially predicted because of short contigs: it is possible that exons of a single gene are predicted on separate contigs; thus, many genes may be double-counted. Nevertheless, the GO categories of predicted genes are comparable to those of other algae, suggesting that major functional genes are contained in the current gene set of *P. yezoensis*. Concerning the rough estimate of gene number, a positive correlation with genome size may be a clue to the argument. If the draft sequences cover most of the *P. yezoensis* genome, the total number of genes is around 10,000 according to the correlation. Sequence homology searching showed that about 35% of the predicted genes have no known function, and around 2,400 gene groups are *P. yezoensis*-specific compared to *C. merolae*, *C. reinhardtii* and *A. thaliana*. Some of those may provide clues about the uncharacterized molecular basis of the lifecycle of *P. yezoensis* or other red algae. Moreover, only 2,069 gene groups are common to *C. merolae*, which is consistent with that these red algae are deeply divergent from each other [50]. Considering that *C. merolae* has adapted to extreme environmental conditions, this unicellular eukaryote may have undergone specific gene gain or loss. In particular, we identified two types of methionine synthases (METH and METE) in *P. yezoensis*: *C. merolae* does not have a *METH* gene and its *METE* gene is of uncertain origin [51]. In hot springs, where vitamin B_12_-producing bacteria are rare, *C. merolae* may not require vitamin B_12_-dependent metabolic pathways. The *P. yezoensis METH* gene will be a target for understanding vitamin B_12_-dependent metabolism and the symbiotic relationship in red algae.

*P. yezoensis* is an important seafood in East Asia and the molecular information is expected to provide a basis for aquaculture. Microsatellite repeats found in this study, mostly CCG triplets, may be useful as polymorphic markers for identifying cultivars. In addition, the photosynthetic system has a critical role in the color of nori, and the related genes may be targets for breeding cultivars resistant to color fading. The R-phycoerythrin γ-subunit gene was already known in other red algae, such as *Aglaothamnion neglectum* and *Corallina officinalis*, but was unreported in *P. yezoensis*. As a whole, we identified phycobilisome-related genes rather than chlorophyll-related genes, suggesting a potential importance in color traits of red algae. In relation to nori aquaculture, a disease of *P. yezoensis*, color fading, is caused by the deficiency of nitrogen and phosphorus essential for the normal growth. In cyanobacteria, nutrient starvation triggers phycobilisome degradation and a small protein plays a key role by being upregulated during the process. The protein, named NblA (non-bleaching A) [52], can bind the phycobilisome complex, though its detailed function is not known. In the case of *P. yezoensis*, an *NblA* homolog (*Ycf18*) was identified from the plastid genome and its expression pattern was investigated, but no induction occurred during the phycobilisome degradation in nitrogen-deficient medium [46]. The same study showed that the upregulation of Ycf18 was induced in ammonium medium, but not in nitrate medium. In the *P. yezoensis* nuclear genome, we found a second homolog of *NblA*, which is evolutionary distinct from the plastid-encoded version. Interestingly, the nuclear *NblA*-like gene has a high GC content (65.0%) and has an intron that interrupts two conserved regions. Therefore, it is not likely that this gene originated through recent horizontal transfer from the chloroplast or cyanobacteria. If this gene has been maintained in the red algal nuclear genome for a long time, it must have an important role in the lifecycle of *P. yezoensis*. Thus, we propose a hypothesis in which the nuclear *NblA*-like gene may be involved in phycobilisome-dependent light harvesting and color fading in nori farms. Otherwise, this gene might affect the growth of *P. yezoensis* in nitrate medium. This hypothesis will be tested by further genome sequencing of other red algae and by expression analysis.

### Conclusions

We determined, for the first time, the symbiont-free genome sequence of *P. yezoensis*. The result suggests that the nuclear genome of this alga is much smaller than previously estimated. The paucity and shortness of the introns within genes support this estimate: intron length is linearly correlated with genome size among algal genomes. This report will call for a rethink of the estimation of red algal genome sizes. From the draft sequence, we obtained important findings about *P. yezoensis* that may be applicable to other red algae. We identified a cobalamin-dependent methionine synthase that has never been reported in red algae. This gene is a promising target for analyzing the interaction with symbiotic bacteria in marine environments. We further detected phycobilisome-related genes in the nuclear genome, some of which may be candidates DNA marker for cultivar improvement. Among these, we report a second homolog of *NblA* in the nuclear genome, and hypothesize that the gene is involved in a color trait of *P. yezoensis*. These findings will shed light on unexplained but probably characteristic features of marine red algae. The *P. yezoensis* genome could represent a model genome for examining red algal life history, and will provide insights into nori aquaculture in the near future.

## Supporting Information

Figure S1 **Read coverage for each contigs.** The dots represent the distribution of contig length with contig coverage (average depth of mapped reads), and histogram shows the frequency of the coverage of contigs. Black and open arrowheads indicate the points of average coverage and the mode of contigs respectively. Black arrow indicates the contig derived from two plasmids (pPY1-U51 and pPY2-U51) (Text S4).
(EPS)

Figure S2 **Microsatellite distribution in the** ***C. merolae*** **genome.** Microsatellite repeats of 2–5 bp unit were detected by Tandem repeats finder, with the same parameters as those for the *P. yezoensis* genome. Repeats with frequency > = 3 are shown.
(EPS)

Figure S3 **Structure of a genomic contig containing a putative gene from γ-proteobacteria.** An 1874-bp contig, which contained a gene similar to the 2-octaprenyl-6-methoxyphenol hydroxylase (*ubiH*) gene of γ-proteobacteria (e.g., GenBank accession number EGU43500), was obtained from the assembled *P. yezoensis* genome data. This contig also contained a transcription elongation factor 1 (*ELF1*) gene of *P. yezoensis* (GenBank accession number; AB480827). The accuracy of the contig assembly was verified by inverse PCR and Sanger resequencing. Arrows indicate gene orientation.
(EPS)

Figure S4 **A detailed Venn diagram of gene sets among** ***P. yezoensis*** **,** ***C. merolae*** **,** ***C. reinhardtii*** **, and** ***A. thaliana*** **.** Numbers of gene groups are shown in black on the diagram. Each gene group is defined as a singleton or a cluster of paralogs. Total numbers of genes included in the groups are shown in red (P, *P. yezoensis*; Cm, *C. merolae*; Cr, *C. reinhardtii*; A, *A. thaliana*).
(EPS)

Table S1 **Species used for gene prediction and comparison.**
(XLS)

Table S2 **Characteristics of algal genes.**
(XLS)

Table S3 **List of public sequences used for the METH tree.**
(XLSX)

Table S4 **List of public sequences used for the NblA tree.**
(XLS)

Text S1 **Treatment of bacterial sequences in protoplast culture.**
(DOC)

Text S2 **Preparation of cDNA library.**
(DOC)

Text S3 **Analysis of gene structure and expression of the** ***P. yezoensis*** **nuclear** ***NblA.***
(DOC)

Text S4 **Plasmids of** ***Pyropia yezoensis*** **.**
(DOC)
