# Supplementary material for: The First Symbiont-Free Genome Sequence of Marine Red Alga, Susabi-nori (Pyropia yezoensis)
Source: PLoS One. 2013 Mar 11;8(3):e57122. doi: 10.1371/journal.pone.0057122 (PMC3594237; doi:10.1371/journal.pone.0057122)

**Treatment of bacterial sequences in protoplast culture**

In the pilot assembly of the *P. yezoensis* genome, we found that some of the contigs obtained were still of organellar (chloroplast and mitochondrion) and bacterial sequences. Regarding the bacterial sequences, we hypothesized that a single species remained in protoplast culture, because a 16S rRNA sequence detected in the contigs was almost identical to that of the genus *Agarivorans*. We determined the genome sequence of *Agarivorans albus* (see below), which was summarized to 80 contigs, and confirmed that most of the remaining bacterial sequences in the *P. yezoensis* sequence were highly homologous to the contigs. Moreover, the whole genome of *Agarivorans albus* was almost covered by the bacterial sequences (Figure S5), strongly supporting our hypothesis. It is most likely that the genomic DNA of *Agarivorans* species is contained in the enzyme solutions for degrading the cell wall of *P. yezoensis*.

Whole genome shotgun sequencing of *Agarivorans albus* mentioned above was carried out by the Roche 454 GS-FLX+ Titanium sequencing platform (Roche Diagnostics, Branford, CT). This bacterium was grown in a 50 ml conical tube with 10 ml of Bacto Marine broth 2216 (Difco) and incubated at 25º C in a shaker incubator for 48 h. The genomic DNA was extracted from the cultured bacteria using the Illustra ™ Bacteria Genomic Prep Mini Spin kit (GE Healthcare). Using the Covaris instrument (Covaris, Inc. Massachusetts, USA), 1μg of the genomic DNA was sheared into 1,500 bp. A 454-pyrosequencing library was constructed from the sheared DNA by GS Titanium Rapid Library Preparation Kit (Roche Diagnostics). Pyrosequencing was performed using 1/4 region of a 70 mm × 75 mm Titanium PicoTiter plate according to the manufacturer's protocols (Roche Diagnostics). The 454 reads were assembled into 80 contigs with Newbler ver. 2.6 (Roche Diagnostics). Alignment of the contig sequences and the bacterial sequences obtained from the *P. yezoensis* protoplast sample was conducted by MUMmer 3.0 [1].

**Reference**

1. Kurtz S, Phillippy A, Delcher AL, Smoot M, Shumway M, et al. (2004) Versatile and open software for comparing large genomes. *Genome Biol* 5: R12.

**Figure S5. Alignment of the *A. albus* genome sequences and the bacterial sequences contained in *P. yezoensis* contigs*.***

The x-axis and y-axis indicate the *A. albus* genome contigs (80 contigs in total) as reference and the bacterial contigs (2520 contigs) obtained in the genome assembly of *P. yezoensis*, respectively.


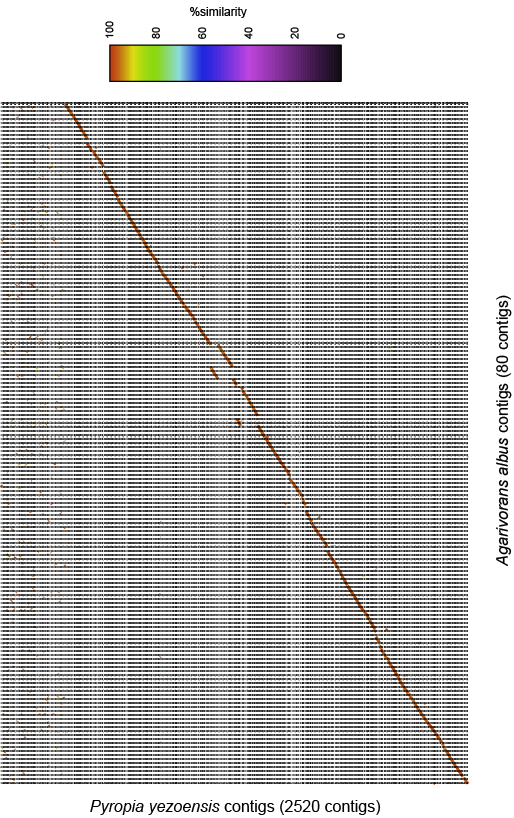

Supplement: Text S1 — Treatment of bacterial sequences in protoplast culture. (DOC) [file pone.0057122.s009.doc]
