# Supplementary material for: The First Symbiont-Free Genome Sequence of Marine Red Alga, Susabi-nori (Pyropia yezoensis)
Source: PLoS One. 2013 Mar 11;8(3):e57122. doi: 10.1371/journal.pone.0057122 (PMC3594237; doi:10.1371/journal.pone.0057122)
Supplement: Text S2 — Preparation of cDNA library. (DOC) [file pone.0057122.s010.doc]

**Preparation of cDNA library**

Total RNA was isolated from thalli of *P. yezoensis* using the RNAqueous kit with Plant RNA Isolation Aid (Life Technologies Corp., Carlsbad, CA, USA). First strand cDNA was synthesized using the GeneRacer kit (Life Technologies) to selectively enrich full-length cDNAs with a cap structure and polyA tail. The cDNAs were amplified by PCR using a proofreading DNA polymerase (KOD-Plus-Neo; Toyobo, Osaka, Japan). The primers used for the PCR were the GeneRacer 5' primer (5'-CGACTGGAGCACGAGGACACTGA-3') and the GeneRacer 3' Primer (5'-GCTGTCAACGATACGCTACGTAACG-3'). The cDNA library was sequenced by Illumina Genome Analyzer IIx.
