# Supplementary material for: The First Symbiont-Free Genome Sequence of Marine Red Alga, Susabi-nori (Pyropia yezoensis)
Source: PLoS One. 2013 Mar 11;8(3):e57122. doi: 10.1371/journal.pone.0057122 (PMC3594237; doi:10.1371/journal.pone.0057122)
Supplement: Text S3 — Analysis of gene structure and expression of the P. yezoensis nuclear NblA. (DOC) [file pone.0057122.s011.doc]

**Analysis of gene structure and expression of the *P. yezoensis* nuclear *NblA***

cDNA was synthesized from total RNA described above by using the PrimeScript RT reagent Kit with gDNA Eraser (Takara Bio Inc., Shiga, Japan) to remove any contaminated genomic DNA. Using this cDNA and protoplast DNA as templates, PCR was performed with KOD-Plus-Neo DNA polymerase (Toyobo, Osaka, Japan).). Gene-specific primers for NblA homolog were designed using the Primer3 web-based tool [1] on the basis of a contig sequence of the *P. yezoensis* genome. The primer sequences were as follows: forward, 5'-TCCGAGCAGCAGTGCAA-3'; reverse, 5'-GCCAGAGAGGAAGTCCGATG-3'. PCR cycles were 94°C for 2 min; 40 cycles of 98°C for 10 s and 68°C for 20 s. PCR products were analyzed using the Agilent DNA 1000 LabChip Kit and the Agilent 2100 bioanalyzer (Agilent Technologies Inc., Santa Clara, CA, USA).

**Reference**

1. Rozen S, Skaletsky H (2000) Primer3 on the WWW for general users and for biologist programmers. *Methods Mol Biol* 132: 365-386.
