# Supplementary material for: The First Symbiont-Free Genome Sequence of Marine Red Alga, Susabi-nori (Pyropia yezoensis)
Source: PLoS One. 2013 Mar 11;8(3):e57122. doi: 10.1371/journal.pone.0057122 (PMC3594237; doi:10.1371/journal.pone.0057122)

**Plasmids of *Pyropia yezoensis*.**

We found two putative complete plasmids, named pPY1-U51 and pPY2-U51, in assembly of the *P. yezoensis* genome (Figure S6, the figure was generated by DNAPlotter [1]). These plasmids are 1,871 and 1,750 bp long, respectively. The GC contents (pPY1-U51; 39.0% and pPY2-U51; 36.2%) were smaller than that of the *P. yezoensis* nuclear genome, rather close to those of organelle. Gene annotations driven by MiGAP [2] showed that pPY1-U51 carries a homolog of a replication-associated protein in *Pyropia tenera* plasmid pPT4-NU (GenBank accession No. FJ956744). Similarly, pPY2-U51 carries a homolog of a putative replicase in *P. tenera* plasmid PT4 (GenBank accession No. AF367410). The whole sequences of pPY1-U51 pPY2-U51 are also similar to those of pPT4-NU and PT4, respectively.

**Reference**

1. Carver T, Thomson N, Bleasby A, Berriman M and Parkhill J. (2009) "DNAPlotter: circular and linear interactive genome visualization" Bioinformatics 25;1;119-20.

2. Sugawara H, Ohyama A, Mori H and Kurokawa K. (2009) "Microbial Genome Annotation Pipeline (MiGAP) for diverse users" The 20th International Conference on Genome Informatics (GIW2009) Poster and Software Demonstrations (Yokohama), S001-1-2.

**Figure S6. Circular map of two *Pyropia yezoensis* plasmids.**

Schematic diagram of pPY1-U51 (A) and pPY2-U51 (B); internal ring represents the GC plot and gray arrows represent the location and the direction of putative protein-coding sequences.


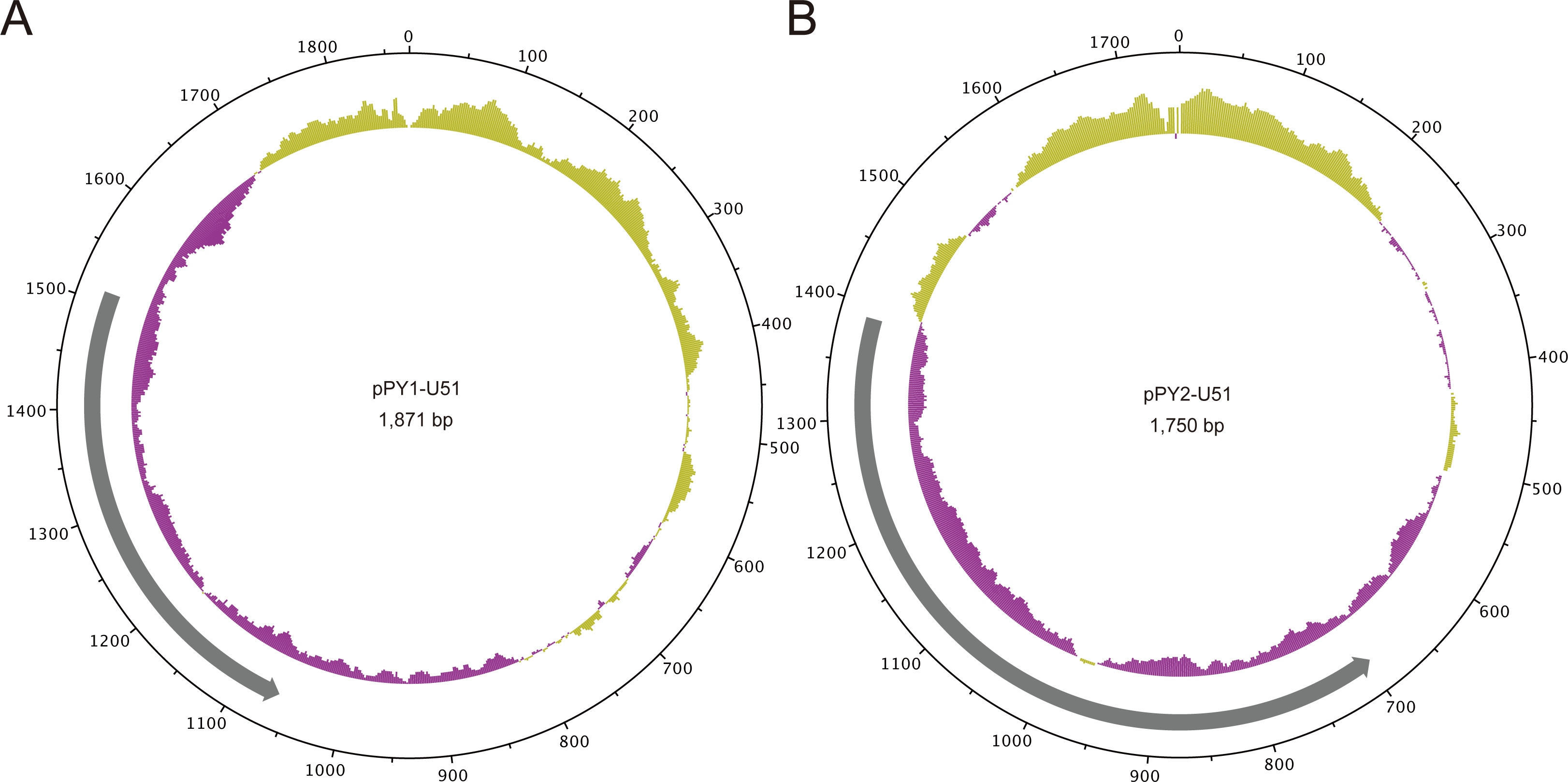

Supplement: Text S4 — Plasmids of Pyropia yezoensis . (DOC) [file pone.0057122.s012.doc]
